# Effects of Humidification on Bran Layer Mechanics and Microstructure of Brown Rice: Mechanism and Optimization

**DOI:** 10.3390/foods15050875

**Published:** 2026-03-04

**Authors:** Yadong Zhu, Zhongqiu Mu, Yifan Lu, Xiangyi Meng

**Affiliations:** 1 College of Mechanical Engineering, Jiangsu University of Science and Technology, Zhenjiang 212100, China; 2 Nanjing Institute of Agricultural Mechanization, Ministry of Agriculture and Rural Affairs, Nanjing 210014, China

**Keywords:** brown rice, humidification, bran layer, mechanical properties, response surface methodology

## Abstract

Humidification conditioning has been increasingly applied in brown rice milling to improve processing performance. However, the underlying mechanisms by which humidification alters the mechanical behavior and microstructure of the bran layer remain insufficiently understood. In this study, the effects of humidification on the mechanical properties and surface microstructure of the brown rice bran layer were investigated, and the optimal conditioning parameters were further determined based on milling performance. Brown rice samples were conditioned to different moisture levels, and the corresponding changes in bran layer tensile strength, surface roughness, and microstructural features were analyzed using tensile testing, three-dimensional surface profilometry, and scanning electron microscopy. The results show that humidification significantly disrupts the continuity of the fibrous matrix in the bran layer, leading to reduced tensile strength and wear resistance. Moderate humidification (around 16% moisture content) promotes the formation of micro-pores and weakens structural integrity, facilitating bran removal during milling and improving head rice yield (*HRY)*, whereas excessive humidification results in over-softening and increased kernel breakage. On this basis, a quadratic orthogonal rotatable composite design was employed to optimize the combined effects of moisture content, humidification time, and equilibration time on *HRY* and specific energy consumption. The optimal conditioning parameters were identified as 16% moisture content, 30 s humidification time, and 36 min equilibration time. This study provides the mechanistic insights into the humidification-induced structural and mechanical evolution of the brown rice bran layer, through experimental optimization of humidification operating parameters, offering practical guidance for improving milling quality and energy efficiency.

## 1. Introduction

Rice (*Oryza sativa*), which serves as a staple food for over half of the global population, needs to go through a series of processes to become edible [1,2]. Brown rice, obtained by dehusking paddy, comprises three main components: the bran layer (6–7% of total mass), the embryo (2–3%) and the endosperm (approximately 90%) [3,4]. Rice milling is a critical processing step that involves removing the bran layer to produce edible white rice [5,6]. In conventional milling, the hard and compact bran layer often requires high mechanical pressure for removal, leading to increased rice breakage, reduced head rice yield (*HRY*) and elevated energy consumption [7]. This conflicts with the growing industrial emphasis on high quality, energy efficiency, and excellent milling uniformity in rice processing. To address these challenges, humidification conditioning has been introduced as a pre-milling treatment. This technique promotes moisture penetration into the bran layer, softening structural components such as cellulose and thereby enabling effective bran removal under reduced milling pressure [8,9,10]. Ultimately, it aims to simultaneously improve *HRY* and lower energy consumption. Therefore, systematically investigating how humidification conditioning influences brown rice milling performance and how optimizing its key process parameters holds significant importance for advancing technology and supporting sustainable development in the rice milling industry.

To reduce the breakage of brown rice during milling, researchers have been committed to improving the efficiency of rice mills in order to minimize milling losses. Poonam et al. [11] indicated that in a friction rice mill, brown rice grains are compressed against each other and pressed tightly against the surface of the rice sieve under the action of the roller shaft. During this process, the frictional forces generated between the rice grains and between the rice and the sieve effectively remove the bran layer from the brown rice. Kalpanadevi et al. [12] also noted that during the milling process, brown rice is pressed against the sieve under mechanical pressure, thereby facilitating the removal of the bran layer. Zhang et al. [13] found that a rice milling roller speed of 1200 rpm was identified as optimal for effectively reducing brown rice breakage and maintaining good milling performance. Furthermore, our previous findings have demonstrated that the structure of the rice mill is a key factor influencing the effectiveness of bran layer removal [14,15,16]. Nevertheless, the mechanism underlying bran layer removal is not yet fully elucidated, necessitating further studies to optimize brown rice milling. The discrete element method (DEM), a numerical technique for analyzing the kinematic and mechanical behavior of discrete particle systems, has proven to be an effective tool for gaining new insights into the phenomena occurring within food processing equipment. The DEM has been applied to investigate the motion characteristics of brown rice particles during the milling process [17,18,19], thereby advancing the understanding of bran layer removal mechanisms. This facilitates the optimization of rice mill structure and operational parameters [20,21]. Although modifying mechanical structures can improve milling performance, such approaches have reached a bottleneck. Fortunately, research indicates that the mechanism of bran layer removal in rice mills includes two aspects: rice milling equipment and brown rice properties [22]. Therefore, it is necessary to improve traditional mechanical processing methods from the perspective of brown rice properties. Shen et al. [23] analyzed the breakage characteristics of brown rice under different moisture contents and stress conditions through confined compression tests, proposed a breakage rate model, and identified four main breakage modes. The results indicate that the loss of cohesive force between starch granules is the micro-mechanism of brown rice breakage during milling. Meng et al. [24] established an experimental platform for the wear characteristics of single brown rice grains and, combined with DEM simulations, investigated the effects of normal force, abrasive particle size, and sliding distance on the wear characteristics of brown rice. They proposed a method for predicting wear rate by integrating wear characteristics with simulation information. The results show that milling with brown rice in a lying position is more conducive to preserving the nutritious embryo. However, this does not fundamentally solve the problem of breakage caused by excessive milling pressure during bran layer removal. Zhang et al. [25] found that humidifying brown rice can effectively soften the bran layer, reduce the pressure required for milling, enhance milling performance, and decrease energy consumption. Therefore, it is of great significance to deeply understand the potential mechanism by which humidification reduces the strength of the bran layer. Li et al. [26] found that the removal of the bran layer during milling mainly includes two forms: wear and peeling. Based on the hypothesis that humidification weakens the wear resistance and tensile strength of the bran layer, this study investigates these mechanical changes from a microscopic perspective and optimizes the humidification process parameters through combined experiments.

This study focuses on investigating the changes in the wear and tensile strength of the bran layer following humidification conditioning. Initially, brown rice was humidified to different moisture contents to evaluate the effects of this process on the surface microstructure of the particle and mechanics of the bran layer. Subsequently, brown rice moisture content, humidification time, and equilibration time were selected as the key factors through preliminary experiments. Using *HRY* and specific energy consumption as the evaluation indices for process performance, a quadratic orthogonal rotation combination experimental design was employed. Data processing and regression analysis were performed using Design-Expert 11 software to reveal the influence patterns of each parameter on the humidification conditioning effect. By establishing a mathematical model between the influencing factors and performance indices, the significance of the key parameters was identified. Ultimately, the optimal combination of parameters yielding the highest *HRY* and the lowest unit energy consumption was obtained, providing a theoretical basis for the optimization of the brown rice humidification process.

## 2. Materials and Methods

### 2.1. Experimental Materials

The rice used in the experiment was japonica rice, variety Dongnong 429, sourced from the Rice Research Institute of Northeast Agricultural University (Harbin, China). Prior to the tests, the paddy rice was subjected to husking using a husking machine, which removed the hulls in a single pass. The resulting product was then cleaned and separated using a whole–broken-rice separator. Subsequently, immature grains, chalky grains, and other defective kernels were manually removed to obtain raw brown rice. The cleaned brown rice samples were packaged in food-grade sealed bags and stored in a refrigerator at 4 °C to prevent moisture absorption from the environment. Before testing, the samples were taken out of the refrigerator and allowed to equilibrate at room temperature for 12 h. The initial moisture content of the brown rice was measured to be 12.1 ± 0.1%.

### 2.2. Experiment Method

#### 2.2.1. Determination of *HRY*

**Figure 1:**
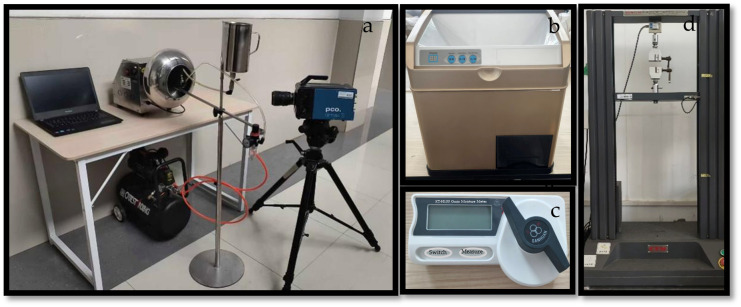
(**a**) Brown rice humidification experimental platform; (**b**) rice mill; (**c**) grain moisture meter; (**d**) universal tensile tester.

20 g of brown rice was weighed each time using an electronic analytical balance (AB204-S, Shanghai, China) and evenly spread in a Petri dish. The samples were uniformly sprayed with a spray device to achieve target moisture contents of 14%, 15%, 16%, 17%, and 18% after humidification conditioning. The moisture content was measured using a grain moisture meter (KT-H100 type; Wuhan Sanhuan Innovation Technology Co., Ltd., Wuhan, China), as shown in Figure 1. After treatment, the brown rice was stood in the same Petri dish for an identical duration before milling. An experimental abrasive-type rice milling machine (YL-M003 type; Zhejiang Yunliang Technology Co., Ltd., Lishui, China) was used for the milling process, as shown in Figure 1. The milling performance of brown rice can be quantitatively evaluated by *HRY*. According to the definition, head rice refers to milled kernels whose length is at least 3/4 of the original brown rice length. Before milling, the initial mass of brown rice was weighed. After milling, the head rice and broken rice were separated using a broken-rice separator, and the mass of the head rice was then weighed. The *HRY* is given by the following formula [27]:

(1) $$HRY\;(\%)=\frac{M_{h}}{M_{i}}\times 100\%$$

where *HRY* denotes the head rice yield, *M_h_* represents the mass of head rice and *M_i_* represents the mass of the initial brown rice. To minimize experimental error, 4 g of milled rice was randomly taken from each group to calculate the *HRY*. The measurement was repeated three times to obtain the average value.

#### 2.2.2. Determination of Specific Energy Consumption

The method for measuring specific energy consumption is as follows. First, a power meter is used to monitor the real-time no-load power of the experimental abrasive-type rice milling machine and the average value is calculated. Then, based on the test conditions of each group, the real-time power during milling and the milling duration are recorded. The total energy consumption for each test condition is obtained through numerical integration. Finally, the specific energy consumption is calculated by dividing the total energy consumption by the mass of brown rice to be milled. The calculation formula is as follows [28]:

(2) $$\begin{array}{c} ES\;(kJ/kg)=\frac{\int_{0}^{t}P_{t}-P_{0}\mathrm{dt}}{M_{b}} \end{array}$$

where *ES* denotes the specific energy consumption; *P_t_* represents the real time power during milling; *P*_0_ is the average real time power under no-load operation of the rice mill; *t* is the milling time and *M_b_* refers to the mass of brown rice being milled.

#### 2.2.3. Determination of Microstructure of the Brown Rice Surface

To analyze the structural changes in the bran layer surface under different treatment conditions, the humidified brown rice samples are first subjected to gold sputtering to enhance conductivity. Subsequently, a scanning electron microscope (Gemini SEM 360, Carl Zeiss Microscopy GmbH, Jena, Germany) is used to observe and capture microstructural images of the brown rice surface at different moisture contents. The wear resistance of the bran layer is indirectly evaluated through its surface roughness. For this purpose, a three-dimensional surface profiler (NANOVEA ST400, NANOVEA Inc., Irvine, CA, USA) is employed to scan the bran layer surface, acquiring high-precision 3D topographic data, which are then used for surface roughness analysis and wear performance assessment. The roughness value of brown rice under each moisture content condition is the average value scanned by a 3D surface profiler in several representative areas (usually 5–8 different views) selected from a single brown rice particle surface system.

#### 2.2.4. Measurement Method for Tensile Strength of the Bran Layer

**Figure 2:**
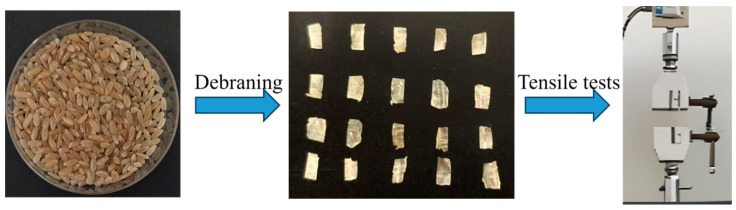
Tensile test of the bran layer.

To evaluate the tensile strength of the bran layer under different treatment conditions, a universal testing machine is used to conduct tensile tests on the bran layer of humidified brown rice. Before testing, the intact bran layer must be separated from the endosperm. The specific procedure is as follows: first, both ends of the brown rice kernel are trimmed off. Then, the kernel is carefully cut along its dorsal side with a blade, and the endosperm is gently removed, leaving a complete bran layer. The bran layer is cut longitudinally into rectangular specimens measuring approximately 4 mm in length and 2 mm in width. The isolated bran layer is shown in Figure 2. To ensure stable gripping in the universal testing machine, both ends of the bran specimen are adhered to paper strips using glue and the specimen is stretched along its longitudinal axis, as shown in Figure 2. This approach was adopted in prior research and demonstrated satisfactory performance [29]. The crosshead speed of the universal testing machine is set to 0.5 mm/min to ensure steady force application. To ensure accuracy, each test condition was replicated 10 times.

### 2.3. Quadratic Orthogonal Rotatable Composite Experimental Design

The experiment was designed by adopting the quadratic orthogonal rotatable composite experimental method, determining the selection range of factors through pre-experiments and the factor level coding table which is shown in Table 1.

### 2.4. Data Analysis Method

The experimental data from the rice milling tests are analyzed using the experimental design software Design-Expert 11, and a quadratic regression model is established. The trapezoidal numerical integration function (trapz) in MATLAB software(2015b) is employed to calculate the specific energy consumption of rice milling according to Equation (2). The *t*-test method in SPSS software (2022) is applied to analyze the significance between the experimental results obtained from the optimized trials and the predicted values.

## 3. Results

### 3.1. Effect of Brown Rice Moisture Content on Milling Performance

**Figure 3:**
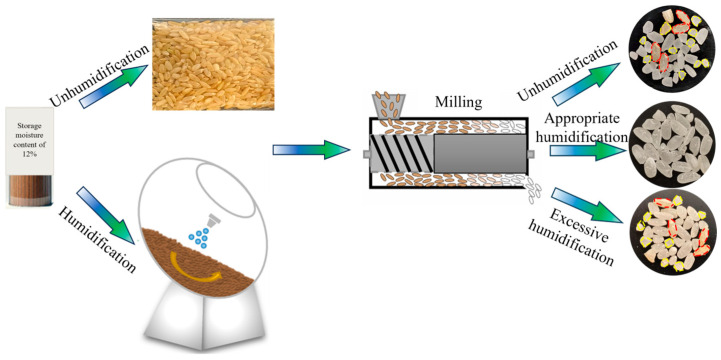
Effect of different moisture contents of brown rice on milling quality.

Figure 3 compares the milling results of humidified and non-humidified brown rice. As shown in Figure 3, through the observation of milled rice grains, it is found that there were milled rice, incomplete milled rice (red dotted line) and broken rice (yellow dotted line). The results show that with the increase in moisture content of brown rice, the milling quality of brown rice will increase first and then decrease. This difference may be due to the fact that excessive humidification of brown rice may affect the change in bran structure, thus leading to the decline in milling quality. Therefore, it is necessary to further conduct a quantitative evaluation of the impact of humidification conditioning on brown rice milling quality in order to define the range of excessive humidification.

**Figure 4:**
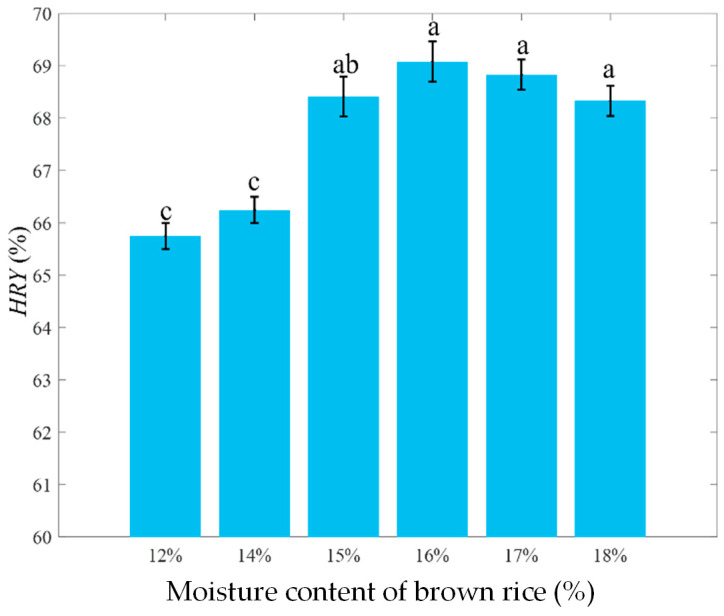
*HRY* of brown rice with different moisture contents. Different lowercase letters above the columns represent significant differences (*p* < 0.05).

To quantitatively evaluate the effect of humidification conditioning on the milling quality of brown rice, the *HRY* under different moisture contents is analyzed. As shown in Figure 4, as the moisture content of brown rice gradually increases, the *HRY* showed a trend of rapid increase at first and then slow decrease. This indicates that humidification conditioning effectively improves milling performance, reduces broken rice, and thereby enhances the integrity and economic value of the finished product. The highest *HRY* was observed at 16% moisture content. Beyond this point, although a slight numerical decrease in *HRY* was noted at 17% and 18% moisture content, statistical analysis revealed no significant difference among these three moisture levels (16–18%), suggesting that *HRY* remains relatively stable within this upper range. This trend implies that moderate humidification helps improve the bonding state between the bran layer and the endosperm, reducing mechanical damage during milling. In contrast, excessive moisture may weaken the structural strength of the bran layer, potentially increasing the susceptibility of brown rice to breakage under mechanical stress. Since the removal of the bran layer during milling primarily occurs through abrasion and peeling, it is necessary to further investigate the mechanism of humidification conditioning from these two perspectives.

#### 3.1.1. Effect of Moisture Content on Surface Microstructure

**Figure 5:**
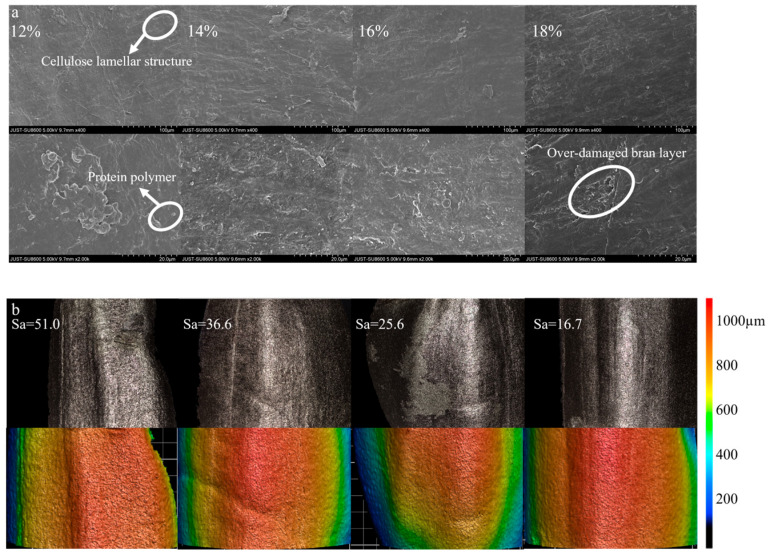
(**a**) Effect of different moisture contents of brown rice on brown rice surface microstructure; (**b**) effect of different moisture contents on the surface roughness of brown rice.

To elucidate the influence of moisture content on the wear resistance of the bran layer, a systematic examination of structural alterations in brown rice under varying humidification conditions was conducted. Since the mechanical properties of the bran layer are intrinsically linked to its microstructure, in this study, scanning electron microscopy (SEM) was employed to characterize the surface morphology of the bran layer at magnifications of 400× and 2000×. Surface roughness was concurrently assessed to complement the microstructural evaluation and provide insight into wear-related changes. As shown in Figure 5a, unhumidified brown rice displays a well-defined, dense, and layered cellulose structure, which contributes to its high mechanical strength and stability, thereby offering robust morphological protection. Following humidification, surfaces of brown rice at initial moisture levels (approximately 12%) exhibit abundant blocky and spherical deposits, largely composed of protein polymers and starch granules [30]. These features correspond to elevated surface roughness (Figure 5b), resulting in an irregular topography that may increase friction and localized stress during milling, ultimately elevating the risk of grain breakage. With increasing moisture content, surface deposits gradually diminish, accompanied by a marked reduction in roughness. Concurrently, the emergence of pores and micro-cracks within the bran layer indicates disruption of the continuous fibrous matrix. This structural degradation compromises the integrity and mechanical strength of the bran layer, thereby reducing its resistance to abrasive wear during milling [31]. However, excessive humidification (e.g., 18%) promotes the formation of an overly porous microstructure, which significantly weakens the bran layer and heightens its susceptibility to fracture.

#### 3.1.2. Effect of Moisture Content on the Tensile Properties of the Bran Layer

**Figure 6:**
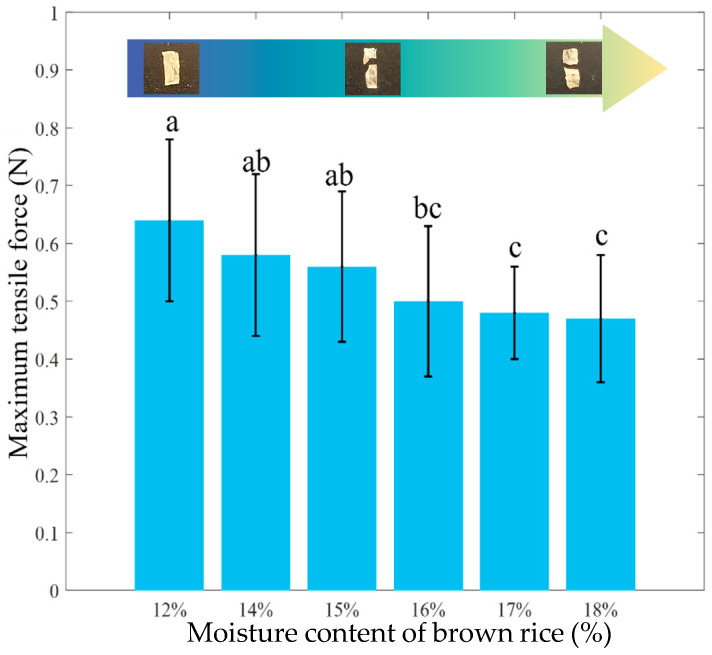
The maximum tensile force exerted on the bran layer of brown rice with different moisture contents. Different lowercase letters above the columns represent significant differences (*p* < 0.05).

Based on the aforementioned findings, humidification treatment significantly reduces the wear resistance of the rice bran layer. During milling in a friction-type rice mill, the bran layer is removed through two main mechanisms: abrasion and peeling. This indicates that, in addition to abrasion, the bran layer also undergoes tensile failure during the milling process. Therefore, this study further analyzes the tensile characteristics of the bran layer after conditioning at different moisture contents. The bran layer is stretched along its longitudinal axis until fracture. As shown in Figure 6, the maximum tensile force sustained by the bran layer decreased progressively with increasing brown rice moisture content, indicating that humidification conditioning markedly reduces its resistance to tensile deformation. To further elucidate the underlying microstructural changes, the fracture morphology of the bran layer was examined across different moisture levels. A clear distinction was observed in the fracture behavior: the high-moisture bran layer failed in a brittle manner with minimal deformation, while the low-moisture bran layer exhibited evident plasticity prior to fracture. The mechanism behind this phenomenon lies in the composition of the bran layer, which consists mainly of cellulose and lipids. This structure provides the rice with good toughness and mechanical properties [32]. However, moisture infiltration disrupts the continuous skeletal structure formed by cellulose and hemicellulose, leading to a decline in the overall toughness and mechanical strength of the bran layer. It is noteworthy that when the moisture content rises to about 16%, the rate of decrease in peak tensile force slows significantly. This is likely because, under the given conditioning parameters (such as temperature and time), the moisture absorption of the bran layer approaches saturation [33]. Beyond this point, further increases in moisture content result in limited additional water uptake, causing the decline in tensile strength to gradually stabilize.

### 3.2. Quadratic Orthogonal Rotatable Composite Experimental Results

Response surface methodology is used to analyze the influence patterns of the three factors on the indicators. A total of 23 sets of experiments are conducted, among which groups 1 to 14 are factorial experiments, and groups 15 to 23 are central experiments for estimating experimental error. The experimental results are presented in the table.

Based on the experimental results in Table 2, regression analysis of the rice milling evaluation indicators is performed. All indicators are modeled using a second-order polynomial model. The expression of the model is as follows:

(3) $$Y=\beta_{0}+\sum_{i=1}^{3}\beta_{i}x_{i}+\sum_{i=1}^{3}\beta_{\mathrm{ii}}x_{i}^{2}+\sum_{i=1}^{2}\sum_{j=i+1}^{3}\beta_{\mathrm{ij}}x_{i}x_{j}$$

where *Y* represents the rice milling evaluation indicator; $\beta_{i}$, $\beta_{\mathrm{ii}}$ and $\beta_{\mathrm{ij}}$ denote the fitting coefficients for the first-order, second-order, and interaction terms, respectively. $x_{i}$ and $x_{j}$ are the independent variables in the model.

#### 3.2.1. Effect of Parameters on *HRY*

The second-order polynomial regression model for *HRY* in relation to moisture content, humidification time and equilibration time is developed using Design-Expert software 11. The analysis of variance for the model is summarized in Table 3.

The analysis of the variance table indicates that the selected regression model has a *p*-value less than 0.05, demonstrating that the overall model exerts a significant influence on the experimental results and is reliable. Furthermore, the coefficient of determination R^2^ for the model is 0.9799, and the adjusted coefficient of determination is 0.9661. The close agreement between the adjusted and unadjusted coefficients of determination suggests a high correlation between the actual and predicted values, confirming the model’s strong predictive capability.

Regression analysis was performed on the experimental results using Design-Expert software, and the non-significant terms *x*_2_, *x*_1_*x*_2_ and *x*_2_*x*_3_ were removed. The regression equation was (with standard errors of the coefficients in parentheses):

(4) $$y_{1}=69.65_{\left(0.05\right)}+0.35_{(0.04)x_{1}}+0.35_{(0.04)x_{3}}+0.13_{(0.06)x_{1}x_{3}}-0.82_{(0.04){x_{1}}^{2}}\;0.19_{(0.04){x_{2}}^{2}}\;0.21_{(0.04){x_{3}}^{2}}$$

**Figure 7:**
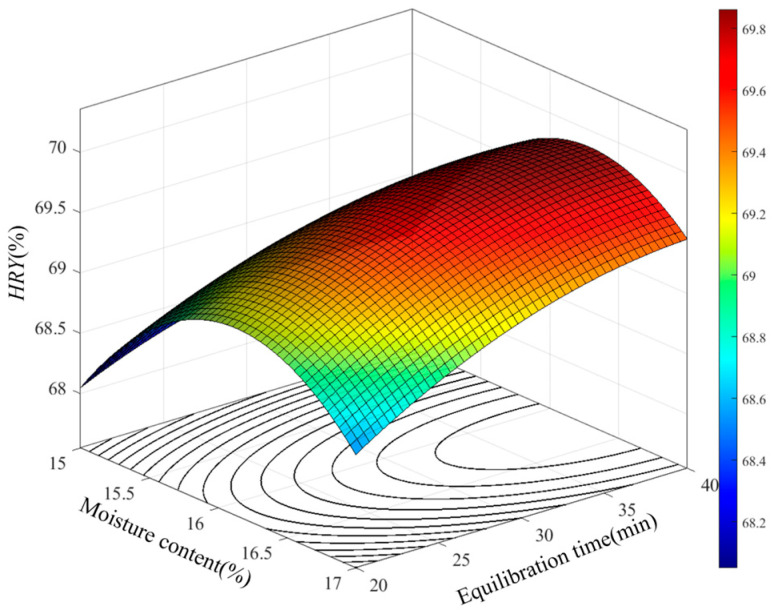
Effect of brown rice moisture content and equilibration time on *HRY*.

The influence patterns of the interaction effects among various factors on the *HRY* were analyzed based on the established regression model. According to the ANOVA results in Table 3, there is a highly significant interaction between brown rice moisture content and equilibration time in the developed *HRY* model. The effect of the interaction between moisture content and equilibration time on *HRY* is illustrated in Figure 7.

The results indicate that when the moisture content of brown rice is at a relatively low level, *HRY* shows a trend of first increasing rapidly and then declining slowly as the equilibration time increases. When the moisture content is relatively high, the *HRY* slightly decreases compared to its peak, suggesting that exceeding a certain moisture threshold is detrimental to milling performance. At different moisture levels, extending the equilibration time has a significant impact on *HRY*, with *HRY* gradually increasing as equilibration time extends, indicating that longer equilibration time helps improve milling quality. This is because prolonged equilibrating softens the bran layer, reducing the pressure required during milling. Consequently, *HRY* reaches its maximum when the humidification time is at its highest level and the brown rice moisture content is at the baseline level.

#### 3.2.2. Effect of Parameters on Specific Energy Consumption

The second-order polynomial regression model for the specific energy consumption in relation to moisture content, humidification time and equilibration time is developed using Design-Expert software. The analysis of variance for the model is summarized in Table 4.

The analysis of the variance indicates that the selected regression model has a *p*-value of less than 0.05, demonstrating that the overall model exerts a significant influence on the experimental results and is reliable. Furthermore, the coefficient of determination R^2^ for the model is 0.9731, and the adjusted coefficient of determination is 0.9545. The close agreement between the adjusted and unadjusted coefficients of determination suggests a high correlation between the actual and predicted values, confirming the model’s strong predictive capability.

Regression analysis was performed on the experimental results using Design-Expert software, and the non-significant terms *x*_2_ and *x*_1_*x*_3_ were removed. The regression equation was (with standard errors of the coefficients in parentheses):

(5) $$y_{2}=26.15_{\left(0.16\right)}-1.08_{(0.13)x_{1}}-1.11_{(0.13)x_{3}}+3.75_{(0.17)x_{1}x_{2}}+0.525_{(0.17)x_{2}x_{3}}+0.85_{(0.12){x_{1}}^{2}}+1.31_{(0.12){x_{2}}^{2}}+1.34_{(0.12){x_{3}}^{2}}$$

**Figure 8:**
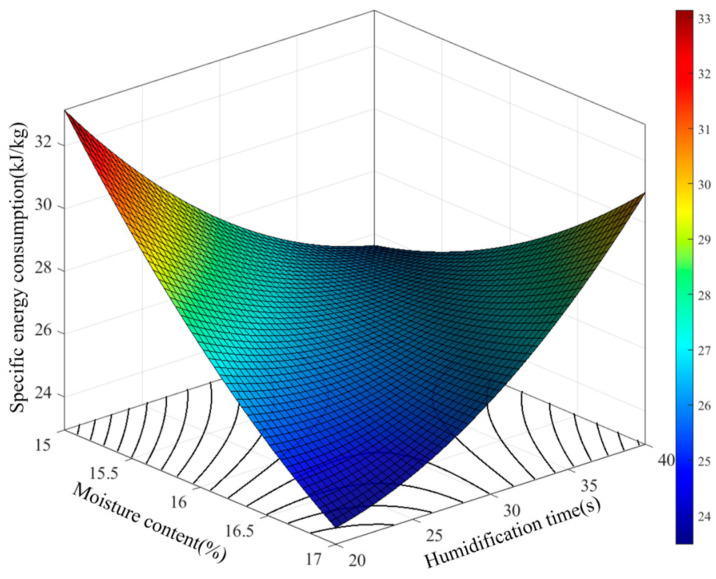
Effect of brown rice moisture content and humidification time on specific energy consumption.

The influence patterns of the interactions among various factors on the specific energy consumption were analyzed based on the regression model. According to the ANOVA results in Table 4, highly significant interactions exist between brown rice moisture content and humidification time, as well as between humidification time and equilibration time, in the established specific energy consumption model. The effect of the interaction between moisture content and humidification time on specific energy consumption is shown in Figure 8.

The results show that humidification time and brown rice moisture content have a significant interactive effect on specific energy consumption. When the humidification time is at a low level, increasing the moisture content first leads to a rapid decrease and then a gradual stabilization in energy consumption. This indicates that even with short humidification, a moderate increase in moisture content effectively softens the bran layer and reduces milling resistance. However, when the humidification time remains at a high level, energy consumption increases with rising moisture content. This is mainly because simultaneously high moisture content and extended humidification cause uneven water distribution on the rice surface. This promotes adhesion between particles, forming dense clusters. Such agglomeration significantly increases the movement resistance of rice in the milling zone, requiring the rice mill to expend more energy to overcome inter-particle adhesion and internal friction. Therefore, within the parameter range of this study, the minimum specific energy consumption occurs when both moisture content and humidification time are at their lowest levels. In practical processing, it is necessary to coordinate the control of humidification time and moisture content, avoiding excessively high levels of both, to achieve effective energy consumption control.

**Figure 9:**
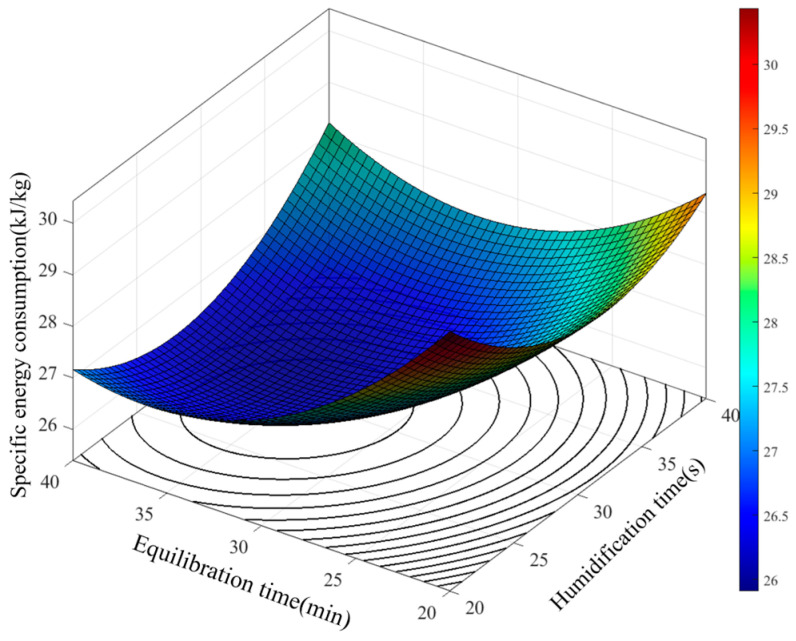
Effect of humidification time and equilibration time on specific energy consumption.

The interaction effect between brown rice humidification time and equilibration time on specific energy consumption is illustrated in Figure 9. The results show that, regardless of the equilibration time level, specific energy consumption first decreases and then increases with the extension of humidification time. When the equilibration time is at a high level, the range of energy consumption values across different humidification times is significantly wider compared to when the equilibration time is at a low level. This indicates that a longer equilibration time leads to a more pronounced decrease in energy consumption when combined with extended humidification. The minimum specific energy consumption is achieved at humidification time of 30 s and equilibration time of 35 min.

### 3.3. Optimization of Humidification Process Parameters

Considering the combined effects of brown rice moisture content, humidification time and equilibration time on milling performance, the humidification process parameters are optimized with the objectives of maximizing *HRY* and minimizing specific energy consumption. The optimization criteria are shown in Table 5. Based on predictions from the regression model, the optimal parameter combination for the humidification process is determined as follows: brown rice moisture content of 16.12%, humidification time of 29.56 s and equilibration time of 36.03 min. In practical production, these parameters are rounded to: moisture content of 16%, humidification time of 30 s and equilibration time of 36 min.

Validation experiments are conducted under the optimal parameter combination, and the results are presented in Table 6. It can be observed that the experimentally obtained *HRY* and specific energy consumption are close to the model-predicted values, with relative errors all below 5%. This indicates that the established quadratic regression model possesses high reliability and can be used for optimizing the humidification process parameters in practical production.

## 4. Conclusions

This study investigated how humidification alters the mechanical properties and surface microstructure of the brown rice bran layer and further optimized the process parameters to improve milling outcomes. Tensile tests, three-dimensional surface profilometry, and scanning electron microscopy analysis demonstrated that humidification induces microstructural changes in the bran layer, leading to reductions in tensile strength and wear resistance. Additionally, response surface methodology was applied to model and optimize the combined effects of moisture content, humidification time, and equilibration time on *HRY* and specific energy consumption. The results indicate that humidification disrupts the fibrous matrix, introduces micro-pores, lowers surface roughness, and weakens the bran layer, thereby facilitating its removal during milling. However, excessive moisture leads to over-softening and higher breakage rates. Optimal milling performance was achieved at 16% moisture content, 30 s of humidification time, and 36 min of equilibration time, maximizing 0 while minimizing energy consumption.

Nevertheless, the present work is limited to a single rice variety under laboratory-scale conditions. Future research should further validate the scalability of the optimized parameters in industrial brown rice milling and evaluate the applicability across diverse rice cultivars to enhance generalizability and practical implementation.

## Figures and Tables

**Table 1:** Factor Level Coding.

Level	Moisture Content *x*_1_/%	Humidification Time *x*_2_/s	Equilibration Time *x*_3_/min
−1.682	14	13	13
−1	15	20	20
0	16	30	30
1	17	40	40
1.682	18	47	47

**Table 2:** Experimental results.

No.	Moisture Content (%)	Humidification Time (s)	Equilibration Time (min)	*HRY* (%)	Specific Energy Consumption (kJ/kg)
1	−1	−1	−1	67.67	32.5
2	1	−1	−1	68.43	30.5
3	−1	1	−1	67.96	30.2
4	1	1	−1	68.13	29.4
5	−1	−1	1	68.23	30.2
6	1	−1	1	69.2	26.5
7	−1	1	1	68.33	29.7
8	1	1	1	69.34	27.8
9	−1.682	0	0	66.75	30.5
10	1.682	0	0	67.9	26.7
11	0	−1.682	0	69.06	30.2
12	0	1.682	0	69.22	29.6
13	0	0	−1.682	68.5	32
14	0	0	1.682	69.63	28
15	0	0	0	69.7	26.6
16	0	0	0	69.6	26.5
17	0	0	0	69.5	25.4
18	0	0	0	69.4	26.6
19	0	0	0	70	26.4
20	0	0	0	69.6	26
21	0	0	0	69.5	26.3
22	0	0	0	69.7	25
23	0	0	0	69.8	26.5

**Table 3:** Analysis of variance of regression model (*HRY*).

Source	Sum of Squares	Degree of Freedom	Mean Square	F	*p*
Model	15.63	9	1.74	70.56	<0.0001 **
*x*_1_	1.72	1	1.72	69.80	<0.0001 **
*x*_2_	0.0182	1	0.0182	0.7409	0.4050
*x*_3_	1.69	1	1.69	68.83	<0.0001 **
*x*_1_ *x*_2_	0.0378	1	0.0378	1.54	0.2371
*x*_1_ *x*_3_	0.1378	1	0.1378	5.60	0.0342 *
*x*_2_ *x*_3_	0.0078	1	0.0078	0.3174	0.5828
*x*_1_^2^	10.85	1	10.85	440.66	<0.0001 **
*x*_2_^2^	0.5412	1	0.5412	21.99	0.0004 *
*x*_3_^2^	0.7079	1	0.7079	28.76	0.0001 **
residual	0.3200	13	0.0246		
lack of fit	0.0578	5	0.0116	0.3526	0.8670
Error	0.2622	8	0.0328		
Total	15.95	22			

Note: * indicates a significant difference, *p* < 0.05; ** indicates a very significant difference, *p* < 0.01.

**Table 4:** Analysis of variance for the regression model (Specific energy consumption).

Source	Sum of Squares	Degree of Freedom	Mean Square	F	*p*
Model	104.28	9	11.59	52.34	<0.0001 **
*x*_1_	16.02	1	16.02	72.36	<0.0001 **
*x*_2_	0.9538	1	0.9538	4.31	0.0583
*x*_3_	16.76	1	16.76	75.69	<0.0001 **
*x*_1_ *x*_2_	1.13	1	1.13	5.08	0.0421 *
*x*_1_ *x*_3_	0.9800	1	0.9800	4.43	0.0554
*x*_2_ *x*_3_	2.21	1	2.21	9.96	0.0076 *
*x*_1_^2^	11.39	1	11.39	51.45	<0.0001 **
*x*_2_^2^	27.11	1	27.11	122.48	<0.0001 **
*x*_3_^2^	28.60	1	28.60	129.20	<0.0001 **
residual	2.88	13	0.2214		
lack of fit	0.2358	5	0.0472	0.1428	0.9768
Error	2.64	8	0.3303		
Total	107.16	22			

Note: * indicates a significant difference, *p* < 0.05; ** indicates a very significant difference, *p* < 0.01.

**Table 5:** Optimization criteria of experimental parameters.

Item	Goal	Lower Limit	Upper Limit	Importance
Moisture content (%)	In range	14	18	3
Humidification time (s)	In range	13	47	3
Equilibration time (min)	In range	13	47	3
*HRY* (%)	Maximum	66.75	70	3
Specific energy consumption (kJ/kg)	Minimum	25	32.5	3

**Table 6:** Optimization results.

Evaluation Index	Predicted Value	Experimental Value
*HRY* (%)	69.8	70.2 ± 0.5
Specific energy consumption (kJ/kg)	26.2	25.8 ± 0.6

## Data Availability

The original contributions presented in the study are included in the article, further inquiries can be directed to the corresponding authors.

## Abbreviations

The following abbreviation is used in this manuscript:

*HRY*	Head rice yield
